# Assessment of bio-contaminants during COVID-19 outbreak from the indoor environment of Hail city, Kingdom of Saudi Arabia

**DOI:** 10.6026/97320630017541

**Published:** 2021-05-31

**Authors:** Mohammed Kuddus, Fahmida Khatoon, Mohd Saleem, Sadaf Anwar, Syed Monowar Alam Shahid, Tarig Ginawi, Ashfaque Hossain, Akram Abdullah Malaqi Alnabri, Ziyad Fayez Alshammari, Abdulaziz Mohammed Alrabie, Sami Shayih Jehad Alenazi, Motaz Monif F Alshammari, Mohd Adnan Kausar

**Affiliations:** 1 Department of Biochemistry, College of Medicine, University of Hail, Hail, KSA; 2 Department of Pathology, College of Medicine, University of Hail, Hail, KSA; 3 Department of Medical Microbiology and Immunology, RAK Medical and Health Sciences University, UAE; 4 College of Medicine, University of Hail, Hail, KSA

**Keywords:** Biocontaminants, Bioaerosols, Indoor environment, Air quality, COVID-19

## Abstract

Biocontaminants are minute particles derived from different biological materials. Indoor biocontaminants are associated with major public health problems. In Gulf countries, it is more precarious due to the harsh climatic conditions, including high ambient temperatures and relative humidity. In addition, due to COVID-19 pandemic, most of the time public is inside their home. Therefore, the aim of the study was to determine the load of biocontaminants in the indoor environment of Hail city. The results showed that most of the bacteria are gram-positive and higher in polymicrobial (87.1%) than monomicrobial (62.7%) association. There was no significant association with sample collection time and types of isolates. The most abundant microbes found in all samples were Staphylococcus aureus followed by Bacillus spp. Among Gram-negative bacterial isolates, E. coli was most common in tested indoor air samples. The study will be useful to find the biocontaminants associated with risk factors and their impact on human health in indoor environment, especially during the COVID-19 pandemic. These results indicate the need to implement health care awareness programs in the region to improve indoor air quality.

## Background

It is a well-known fact that biocontaminants are one of the major sources of indoor air pollution. The majority of them include mold,bacteria, dust mites, and other antigens. These biocontaminants play a crucial role in decreasing indoor air quality. Exposure to these airborne biocontaminants can prompt irritation, mild to severe allergic reactions, respiratory disorders, infections, and chemical responses [1,2]. Some of the biocontaminants have the ability to resolve rapidly within the indoor air environment [2], and over time, they may become nonviable and fragmented by the process of dehydration. These nonviable fragments of organisms are common and can be toxic or allergenic, depending upon the specific organism or organism component. Once these smaller and lighter fragments become suspended in the air, they have a greater tendency to stay suspended [2,3]. Bioaerosols are tiny, airborne particles (such as-fungal spores, pollen grains, endotoxins, or particles of animal dander) that are composed of or derivative of biological matter [4]. Bioaerosols come in different sizes, ranging from several nanometers to more than 100 µm, subject to relative change based on humidity, temperature and other climatic factors [5]. Globally the concern with bioaerosol exposure has increased over the past few decades. This is mostly due to the recognition that exposure to biological agents in the indoor environment is associated with a wide range of pathological conditions with major public health burdens ranging from contagious diseases to malignancies [6]. In 2017, about 1.6 million people died prematurely as a result of indoor air pollution [7]. Several studies have indicated that microorganisms of fungal and bacterial genera are very common in residential environments. Cladosporium, Penicillium, Alternaria, and Aspergillus are the most prevalent ones. Recently, exposure to fungal fragments has been of principal importance since they have been recognized as an active factor for respiratory illness and atopic dermatitis [[8]. Bacterial endotoxin has also been suggested to play a crucial role in the development of atopy and asthma in the indoor environment [9]. Coronavirus (SARS-CoV-2) is a very infectious disease. When a human coughs or exhales, tiny droplets from the nose or mouth may transmit the disease from one person to another. People may become infected with the virus by inhaling droplets from an infected person who coughs out or exhales droplets. In Saudi Arabia, indoor air pollution is a human health threat due to the harsh meteorological conditions such as high ambient temperatures, high relative humidity, and natural events like sandstorms force people to spend most of their time indoors [10]. In addition, due to limited and inconclusive findings about the airborne transmission of SARS-CoV-2, researchers are encouraged to implement more studies in this area. Therefore, this study aims to identify microbial aerosols in the indoor air of domestic homes, more specifically in the city of Hail, Kingdom of Saudi Arabia.

## Methodology

### Study area:

The present study was conducted at three different locations (airport area, residential area, college of medicine, University of Hail) of Hail city, Kingdom of Saudi Arabia.

### Study period:

The research was done during the months of October and December in 2020, with an average temperature between 29°C to 9°C and average humidity of 20%.

### Study subject:

The study includes 90 air samples collected from different areas viz. residential areas, airport areas, and College of Medicine, in Hail city, Kingdom of Saudi Arabia.

### Collection of samples:

The air samples were collected on nutrient agar plates. The plates were exposed to an indoor environment for 15-20 min and transported to the lab at room temperature for further processing. The collected samples were incubated aerobically at 37°C for 1-3 days and evaluated for microbial growth.

### Sub-culture and sample purification:

All bacterial growth were sub-cultured to obtain pure culture on Nutrient Agar, Sheep blood agar (5%), MacConkey agar, and Chocolate agar; and incubated at 37°C for 24 hours [11].

### Biochemical analysis and isolates identification:

All the isolated microorganisms with different morphology were identified and characterized by Gram-staining and biochemical tests following Bergey's Manual [12]. The major tests include coagulase, catalase, oxidase, urease, nitrate reduction, lactose fermentation, indole, methy red, Voges-Proskauer, citrate utilization, and Hydrogen sulphide production test [13-14].

### Statistical analysis:

The results are presented in frequencies and percentages. The Chisquare test was used to assess the associations. The p-value <0.05 was considered significant. All the statistical analysis carried out by using SPSS 23.0 version (IBM Inc, Chicago, USA).

## Results:

**Figure 1:**
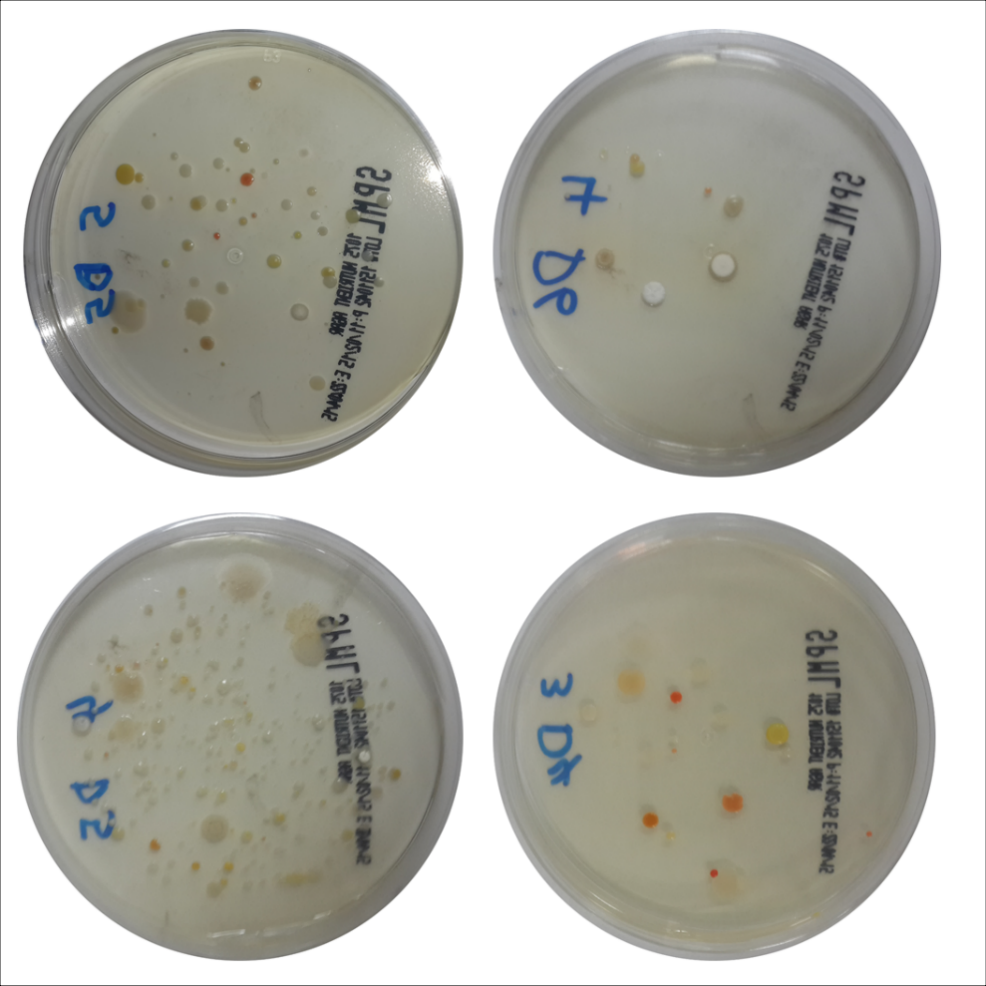
Representative plates of microbiological colony obtained from air samples of different locations

**Figure 2:**
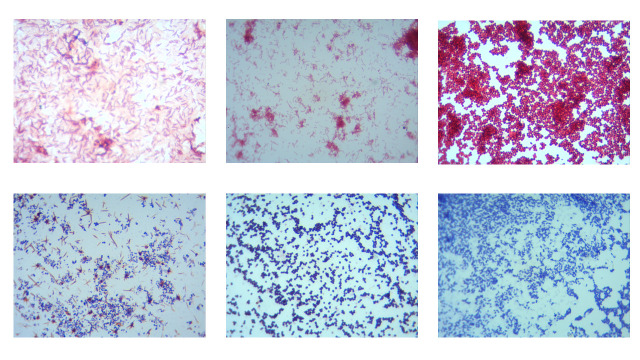
Morphology of bacterial isolates under the microscope

In this study, a total 90 indoor air samples were included (Figure 1). Bacterial isolates were identified and presented in Table 1 (see PDF). Out of the total sample, more than half of the air samples were from the residential area (64.4%), followed by the college of medicine (32.2%) and airport area (3.3%) of Hail city, KSA. 59 samples have monomicrobial growth and 31 show poly-microbial growth in each culture plate. More than half of the isolates (65.6%) in each plate showed single morphology (Figure 1). Gram-positive bacteria were among the majority of samples (71.1%) in all the areas of Hail, KSA (Figure 2, Table 1 and 2 - see PDF).

**Figure 3:**
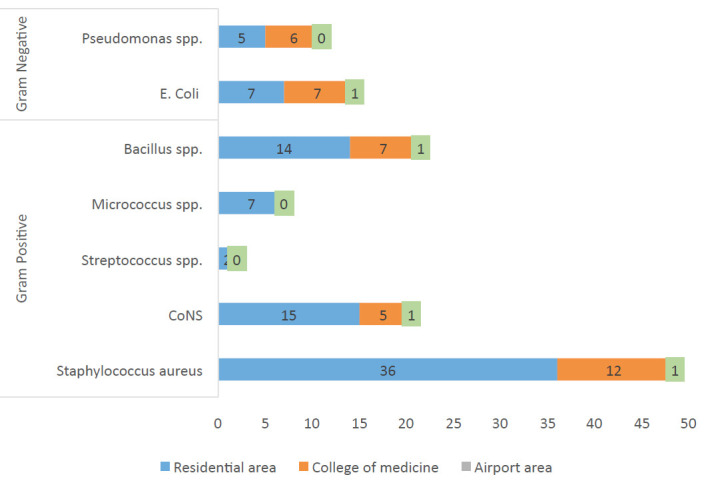
Distribution of bacterial isolates according to different locations

Gram-positive bacteria were most common in residential areas (74.1%) with no significant (p>0.05) association of location with type of bacteria. Gram-positive bacteria were higher in polymicrobial (87.1%) than Monomicrobial (62.7%) with a significant association (p=0.01). There was no significant (p>0.05) association between sample collection time and types of bacterial isolates (Table 3 - see PDF). Among all the isolates, Staphylococcus aureus was dominant (36) in the residential area, followed by the college of medicine (12) and airport area (1). Coagulase-negative Staphylococci, Bacillus spp. and E. coli were isolated only one at the airport area. Pseudomonos spp was common in the college of medicine (6), while it was found to be five in the residential area. In the residential area, only two Streptococcus spp was reported (Table 4 - see PDF and Figure 3). As shown in Figure 3, Staphylococcus aureus was most dominant bacterial species among Gram-positive isolates in the indoor environment, whereas Streptococcus spp was the least. On the other hand, E. coli was highest among gram-negative bacterial isolates in the indoor environment, while Pseudomonos spp was reported in the least number of isolates.

## Discussion:

Nowadays, particularly during the COVID-19 pandemic, people are spending around 90% of their daily time in a closed atmosphere, especially at home. During this period, concentrations of some air pollutants maybe two to five times higher in the indoor environment rather than outdoors. Indoor air pollution is the eighth most important risk factor, which is accountable for 2.7% of the global load of disease [15]. Many disorders have been found to be associated with poor indoor air quality; among which respiratory diseases are the most important as inhalation is a major pathway for air pollutants [16]. Indoor environments may contain various contaminants such as microbes that can negatively affect human health [[17-19]. Grice and Segre (2012) reported that microorganisms restrain human health by various mechanisms and the presence of micro containments or microbes are an important source of genetic diversity and a functional entity that influences metabolic behavior [20]. Indoor microbial air quality is influenced by factors such as air density and quality, hygiene conditions and ventilation system. Since the present study measured the microbial load and identified the presence of microorganisms present in the air of indoor environment, we can compare the diversity of microcontinents identified in all the other regions of the globe concerning numerous factors that influence air quality in indoor environment. Indoor bioaerosols presence might play a crucial role in these regions for the occurrence of endemic conditions. In our study, assessment of biocontaminants level in the indoor environment of various locations in Hail city indicates the presence of major strain of bacteria. However, several bacterial strains were isolated from air by cultivation on nutrient agar media, Grampositive bacteria were found in majority of the samples (71.1%) in all the sampling area of Hail. Moreover, no fungal strains were isolated on agar media. Gram-positive bacterial species were resistant to environmental influence and more prevalent than Gram-negative bacterial species and fungal species. A similar result was found by a study conducted by Bakutis et al. (2004) identified more Gram-positive bacteria in livestock farms and poultry houses [21]. Regarding the Gram-negative bacteria isolated in the study, E coli and Pseudomonas spp have not been associated with major livestock diseases as shown by Bakutis et al. [21]. Our study also suggested that Staphylococcus aureus was dominant in dwellings,followed by educational building (college of medicine) and least in the airport area. Coagulase-negative Staphylococci, Bacillus spp. and E. coli were isolated only one at airport area. Pseudomonos spp was common in education building while it was found to be less in residential area. In residential area, only two Streptococcus spp were reported. In contrast, study conducted in Jordan by Afnan Al-Hunait et al. in 2016 [22] showed that at in the educational building, concentration of the Gram-positive bacteria and Penicillium/Aspergillus spp. were lower than those observed in the residential area. Similarly, quantity of the Gram-negative microbes, at the education building was rose to peak as compare to residential settings. Also reported that total fungi concentrations were same range in educational and the dwellings, although in our study fungi were not studied. They have found that all faculty offices, lecture halls, and the corridors had higher gram-negative bacterial concentrations in comparison to the main entrance corridor. In our study, Staphylococcus aureus was most dominant bacterial species among Gram-positive isolates in indoor environment, whereas Streptococcus spp was the least. On the other hand, E. coli was highest among Gram-negative bacterial isolates in indoor environment, while Pseudomonos spp was reported in least concentration. The differences in bacterial concentrations among the studied indoor environments of residential and airport and educational buildings in this study indicate that sources of this biocontamination are diverse in various locations of Hail city. Similarly, a study conducted in 2014 by some researcher confirmed that indoor microbes that originate from outdoor cradles vary in space and time, which relates to various biological conditions as major factors affecting the concentrations of biocontainment region [23]. Another study by Weikl et al. in 2016 [24] reported that certain environmental conditions, including vegetation's and outdoor particulate matter affect more on fungi than to bacteria species. The small deviations in Gram-positive microbes indoor circumstances could be sanctioned to the positive strains tolerance to these changes like dry weather conditions [25]. In the present study, Gram-positive bacteria showed no significant (p>0.05) association with air samples studied. Data shows that gram-positive bacteria were higher in polymicrobial (87.1%) than monomicrobial (62.7%) with significant (p=0.01) association. There was no significant (p>0.05) association between sample collection time and types of bacterial isolates. Our results agree with previous studies that major sources of micro-contaminants attributed by humans' activities and human occupancy in residential areas [26-28]. Similarly, some other studies also reported that the nature of human contact and human behavior has great influence on in door environment [29,30] for instance, presence of bacteria in the kitchen surface area is influenced by the various kind of food [31], and surfaces area most often affected by hands containing higher concentrations of skin flora [32]. In commercial or social settings, bacteria of outdoor origin may be a more significant source for surfaces, although normal skin flora or microbes are also present in high concentration [33]. The airborne bacteria found indoors also suggest a strong influence of human-associated bacteria as a primary source, in addition to outdoor-associated pathogens [34,35]. The study suggests that the health sectors need to be reevaluated for proper health management, including microbial contamination prevention and control in indoor environments.

## Conclusion:

As evident from the published literatures, indoor air quality can affect human's life and brings life-threatening diseases because it contains biocontaminants, including pathogenic agents such as bacteria, fungi, viruses, toxins and other infectious materials. Biocontaminants could cause a variety of diseases associated with the lungs, intestines, kidneys and central nervous system. At present COVID-19 outbreak also nailing indoor air quality with serious threat to human health. We found that the indoor environment of Hail city, Saudi Arabia had a significant level of microbial load, especially Staphylococcus aureus, Bacillus spp. and E. coli that can cause various health issues in the community. Better ventilation of the indoor environment and community awareness about indoor air quality and its complications is needed to improve quality of life and reduce biocontaminant induced health issues.

## Ethical approval:

The institutional ethical committee approved the study with number 55456/5/41 dated August 19, 2020.
